# RMI2 regulates recombination intermediate metabolism to ensure proper chromosome segregation in mammalian meiosis

**DOI:** 10.1093/nar/gkag950

**Published:** 2026-10-05

**Authors:** Luciana Previato de Almeida, Tolkappiyan Premkumar, Monika K Kawecka, Irma Gryniuk, Agustin Carbajal, Francesca Cole, Roberto J Pezza

**Affiliations:** Cell Cycle and Cancer Biology Research Program, Oklahoma Medical Research Foundation, Oklahoma City, OK 73104, United States; Department of Epigenetics and Molecular Carcinogenesis, Division of Basic Sciences, The University of Texas MD Anderson Cancer Center, Houston, TX 77030, United States; UTHealth Houston Graduate School of Biomedical Sciences, The University of Texas MD Anderson Cancer Center, Houston, TX 77030, United States; Cell Cycle and Cancer Biology Research Program, Oklahoma Medical Research Foundation, Oklahoma City, OK 73104, United States; Department of Cell Biology, University of Oklahoma Health Sciences Center, Oklahoma City, OK 73104, United States; Cell Cycle and Cancer Biology Research Program, Oklahoma Medical Research Foundation, Oklahoma City, OK 73104, United States; Department of Cell Biology, University of Oklahoma Health Sciences Center, Oklahoma City, OK 73104, United States; Cell Cycle and Cancer Biology Research Program, Oklahoma Medical Research Foundation, Oklahoma City, OK 73104, United States; Department of Epigenetics and Molecular Carcinogenesis, Division of Basic Sciences, The University of Texas MD Anderson Cancer Center, Houston, TX 77030, United States; UTHealth Houston Graduate School of Biomedical Sciences, The University of Texas MD Anderson Cancer Center, Houston, TX 77030, United States; Cell Cycle and Cancer Biology Research Program, Oklahoma Medical Research Foundation, Oklahoma City, OK 73104, United States; Department of Cell Biology, University of Oklahoma Health Sciences Center, Oklahoma City, OK 73104, United States

## Abstract

Proper segregation of meiotic chromosomes depends on the controlled formation and resolution of recombination intermediates that give rise to crossovers (COs). While the BTRR complex—comprising the BLM helicase, topoisomerase IIIα, and the proteins RMI1 and RMI2—is a well-characterized regulator of recombination in somatic cells, its function during meiosis remains incompletely understood, particularly for RMI2 in mammals. Here, we investigate the role of RMI2 in mouse spermatogenesis using a targeted RMI2 knockout model. Loss of RMI2 results in severe meiotic defects, including reduced testis size, increased apoptosis at the metaphase–anaphase I transition, and frequent chromosomal abnormalities such as lagging chromosomes in metaphase I cells and anaphase I chromatin bridges. Cytological analyses reveal disruptions in the processing of recombination intermediates. Our work monitoring molecular recombination outcomes at known recombination hotspots suggests that RMI2 regulates the balance of recombination pathways, constrains aberrant branch migration, and ensures proper CO maturation. Our findings show previously unappreciated functions for RMI2 essential for coordinating recombination intermediate metabolism and promoting proper chromosome conformation and segregation during meiosis. These results provide insights into the complex regulation of recombination intermediate homeostasis in higher eukaryotes and highlight the role of RMI2 in maintaining genome stability and proper gamete development in mammals.

## Graphical Abstract

**Graphical Abstract:**
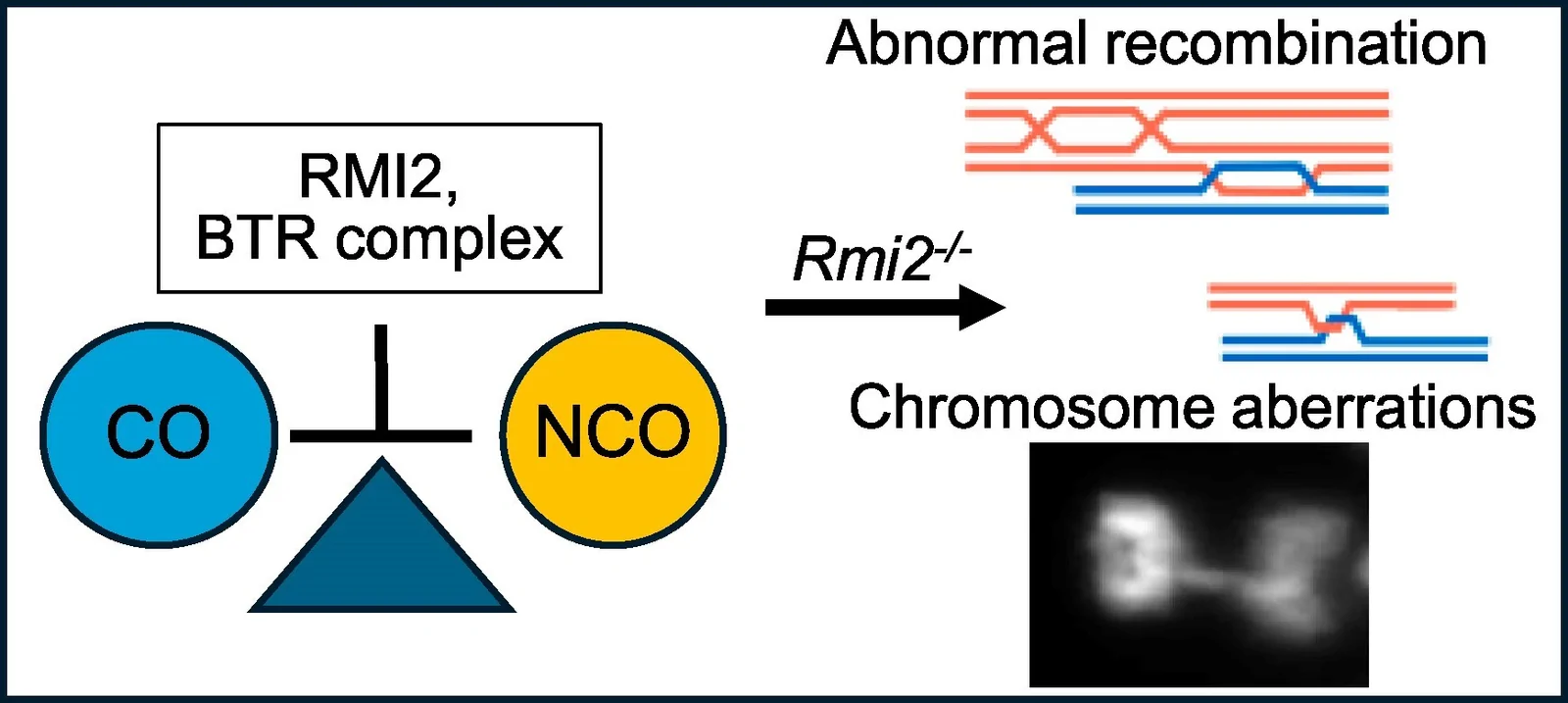

## Introduction

The first meiotic division is crucial in ensuring the proper segregation of chromosomes, where maternal and paternal copies of each chromosome are separated into distinct cells. This process relies on homologous recombination, which is initiated by programmed double-strand breaks (DSBs) and culminates in the formation of crossover (CO). These COs, together with cohesin, hold homologous chromosomes together and orient them correctly on the meiotic spindle, facilitating accurate chromosome segregation [1, 2]. Invariably, failure in the proper formation or resolution of COs leads to chromosome segregation errors, contributing to aneuploidy in gametes. The significance of this process is underscored by the fact that ~10%–30% of human meiocytes experience chromosome segregation errors, highlighting the importance of maintaining accurate meiotic recombination [3, 4].

Meiotic recombination begins with SPO11-induced DSBs, followed by resection of the 5′ ends to expose 3′ single-stranded DNA (ssDNA) overhangs [5]. The ssDNA is bound by the RPA complex, and the recombinases RAD51 and the meiosis-specific DMC1 form a nucleoprotein filament capable of performing a homology search on the intact homologous chromosome [6]. This search results in strand invasion, forming heteroduplex intermediates that can be extended by DNA synthesis. These joint molecule (JM) intermediates are processed by one of two pathways: the non-crossover (NCO) pathway or the CO pathway [7]. Most COs are formed through a process that requires the ZMM protein group [8]. In mouse, this includes SYCP1 [9], HFM1 [10], SHOC1 [11], and the MSH4-MSH5 complex [12], and exhibit the phenomenon of interference, where COs are further apart than expected by chance.

The resolution of JMs must be tightly controlled to ensure the correct balance between CO and NCO formation and to prevent excess of recombination products and/or formation of inappropriate recombination, such as nonallelic recombination, ultimately resulting in defective meiotic chromosome segregation. A variety of JM structures have been identified in organisms undergoing recombinational repair, including canonical structures such as D-loops, extended D-loops, and Holliday junctions, as well as more complex structures, such as multi-chromatid JMs. The regulation of JM processing during meiosis is orchestrated by a network of proteins, with protective activity such as HFM1 [10], SHOC1 [11], and the MSH4-MSH5 complex [12, 13] playing a key role in stabilizing nascent JMs [8, 14] and promoting CO formation through the double Holliday junction (dHJ) resolution pathway by the endonuclease MutLγ (MLH1–MLH3) [15–17]. These activities contrast with the activity of restriction endonucleases to ensure JM resolution such as GEN1, SLX1-SLX4, and MUS81-MMS4 [18–21], and dissolution mediated by the Bloom’s syndrome helicase (BLM) [22].

In mammals, the BLM protein, in conjunction with topoisomerase 3 (TOP3) and the OB-fold proteins RMI1/RMI2, forms the BTRR complex. The deletion of BLM highlights the significant role of the BTRR complex in normal meiotic progression. BLM mutant spermatocytes exhibit severe defects, including improper pairing and synapsis of homologous chromosomes, as well as altered processing of recombination intermediates, leading to increased chiasmata. Consistent with this, studies from various model organisms demonstrate that the BTR complex plays critical roles in the processing and placement of meiotic COs and NCO [23–36]. BLM or its orthologs are primarily responsible for dismantling JMs via decatenation activity [37–39], preventing the formation of aberrant off-pathway meiotic JMs by dissociating promiscuous strand exchange products [36, 40, 41].

RMI2 is the less understood component of the BTRR complex. From studies in somatic cells, RMI2 has been proposed to have a less important role by acting as a scaffold protein, stabilizing the other members of the complex within the recombination sites [42]. However, RMI2 depletion results in spontaneous chromosomal breaks [42], and humans lacking RMI2 have Bloom-like syndrome. In these patients, somatic cells exhibit elevated rates of sister chromatid exchange, anaphase DNA bridges, and micronuclei, suggesting increased recombination and genome instability [43]. Together, the available data indicate a possible unrecognized central role for RMI2 in regulating recombination DNA intermediate metabolism.

Regarding the function of RMI2 in the germline, so far, studies in *C. elegans* have shown that the RMI2 ortholog (RMIF-2) stabilizes RMH-1 (an RMI1 ortholog) and promotes loading of HIM-6 (Bloom helicase) and TOP3 onto recombination foci [44]. Although these results suggest a possible role for RMI2 in regulating recombination in meiotic cells, the molecular mechanism of RMI2 action remains unknown across organisms. Also, we do not know the requirements of RMI2 in meiosis of higher eukaryotes.

To determine the mechanism of RMI2 function in mouse meiosis, we created and characterized RMI2 knockout mice. RMI2 deletion resulted in defects in meiotic cell divisions, accompanied by prominent aberrations in chromosome conformation, such as metaphase I lagging chromosomes and anaphase I chromatin bridges. Our work at the molecular level defines the role of RMI2 and its function within the BTRR complex by showing that RMI2 loss alters gene conversion patterns consistent with defects in specific recombination intermediates, including failures to properly constrain branch migration and form the heteroduplexes required for accurate CO maturation. Contrary to the prevailing view of RMI2 as a passive scaffold, our results suggest that RMI2 plays a direct and decisive role in regulating recombination-pathway choice and maintaining a balance between CO and NCO outcomes. This work establishes an important role for RMI2 in normal mammalian meiosis, extending its functional significance beyond previously studied systems and highlighting its contribution to genome stability.

## Materials and methods

### Mice and genotyping

Experiments conformed to relevant regulatory standards guidelines and were approved by the Oklahoma Medical Research Foundation-IACUC (Institutional Animal Care and Use Committee), Protocol number 17–25. Unless specified otherwise, mice analyzed were 2-months old.

To generate RMI2-deficient mice, we created (TIGM) a parent cell line (ES Cells) with C57BL/6N as the strain of origin. In this strain, loxP sites were inserted between exon 1. The cassette is composed of an FRT-flanked lacZ/neomycin sequence followed by a loxP site. A conditional (floxed) allele can be created by Flp recombinase expression in mice carrying this allele (further details in Supplementary Fig. S1). Transgenic Cre recombinase mouse Ddx4-Cre^FVB-Tg (Ddx4-cre)1Dcas/J^ line was purchased from The Jackson Laboratory (Bar Harbor, ME). Gonad-specific knockouts and wild-type heterozygote littermates were obtained from crosses between female homozygous flox/flox mice with male heterozygous Cre/+; Wt/flox. The gonad-specific-Cre-mediated recombination in germ cells and thus Ddx4-Cre carriers cannot transmit the floxed allele, e.g. Ddx4-Cre; RMI2^Wt/fl^ mice can only transmit the null (−) or wild type *Rmi2* alleles.

Genomic DNA was collected from tail tips digested at 95°C in buffer A (25 mM NaOH, 0.2 mM EDTA) for 60 min, and the reactions were stopped by adding buffer B (40 mM Tris, pH 5.0). The supernatant was collected for downstream PCR (polymerase chain reaction) genotyping with EconoTaq PLUS GREEN 2X Master Mix (Biosearch Technologies, 30033-1), according to the manufacturer’s instructions.

C57BL/6J × DBA/2J F1 hybrid male mice were generated by crossing inbred mouse strains C57BL/6J (B) and DBA/2J (D). Single *Rmi2* or *Blm* mutants were generated from independently maintained heterozygous mice in the B and D strains, which were genotyped to verify homozygosity for the corresponding haplotype at the *59.5* hotspot. *Blm* mutant mouse [22] was a kind gift from Dr Paula E. Cohen.

All mice used in this part of the study were adult males, aged 2–6 months.

### Histology and immunostaining

Testes or ovaries were dissected, fixed in 10% neutral-buffered formalin (Azer Scientific Inc., B-PFNBF-20) and processed for paraffin embedding. After sectioning (5–15 μm), tissues were positioned on microscope slides and analyzed using hematoxylin and eosin or TUNEL assay (Roche) according to standard protocols. For TUNEL assay, slides were subsequently counterstained for 3 min with 2 µg/ml DAPI containing Vectashield mounting solution (Vector Laboratories) and sealed with nail varnish. We use Axiovision SE 64, Zeiss LSM-710 Confocal (Carl Zeiss Inc.) or Nikon C2 Confocal (Nikon) for imaging acquisition and processing.

### Western blot

After testis detunication the samples were sonicated (three pulses of 40 s) in 2× sample buffer [4% sodium dodecyl sulfate, 160 mM Tris–HCl (pH 6.8), 20% glycerol, 4% mM β-mercaptoethanol, and 0.005% bromphenol blue]. Thirty to fifty micrograms of sample was separated by 4%–15% gradient Tris–acetate sodium dodecyl sulfate–polyacrylamide gel electrophoresis (SDS–PAGE) and electro-transferred to a PVDF membrane (Sigma Aldrich, 3010040001). The blots were probed with individual primary antibodies and then incubated with HRP-conjugated antibody as required. In all blots, proteins were visualized by enhanced chemiluminescence, and images were acquired using the Western Blot Imaging System c600 (Azure Biosystems). α-tubulin was used for normalization. Antibodies used are detailed in Supplementary Table S1.

### Chromosome spreads and immunofluorescence

We used established experimental approaches to visualize chromosomes in chromosome surface spreads [45]. Incubation with primary antibodies occurred for 12 h at 4°C in 1× phosphate buffered saline (PBS) plus 2% bovine serum albumin. Slides were incubated with primary antibodies (Supplementary Table S1) followed by three washes in 1× PBS and then incubated for 1 h at room temperature with secondary antibodies. A combination of fluorescein isothiocyanate (FITC)-conjugated goat anti-rabbit IgG (Jackson laboratories) with rhodamine-conjugated goat anti-mouse IgG each diluted 1:250 was used for simultaneous double immunolabeling. Slides were subsequently counterstained for 3 min with 2 μg/ml DAPI containing Vectashield mounting solution (Vector Laboratories) and sealed with nail varnish. We used Axiovision SE 64 (Carl Zeiss) for imaging acquisition, visualization for immunosignal counting, and processing.

### Metaphase spreads

The protocol was performed as previously described [46]. Testes were dissected, decapsulated, and incubated in a 2.9% isotonic sodium citrate solution at room temperature. Following the generation of single cell suspension, cells were transferred to a 15-ml conical tube, avoiding residual tissue fragments, and centrifuged. The pellet was resuspended in 1% hypotonic sodium citrate and incubated for 12 min at room temperature. After a second centrifugation, the cells were resuspended and fixed for 5 min using a solution composed of 200-proof ethanol, glacial acetic acid, and chloroform in a 3:1:0.025 ratio. A second round of fixation was carried out using fresh fixative for an additional 5 min. Approximately 30 μl of the final cell suspension was dispensed onto a microscope slide, allowed to air-dry, and stained for 10 min in DAPI solution or a 1:20 dilution of Giemsa prepared in 1× PBS (pH 6.5) with gentle agitation. Slides were subsequently rinsed with water, dried, and mounted with coverslips for microscopic examination.

### q-PCR

Total RNA was isolated from adult testes (2-months old) with the Direct-zol RNA MiniPrep Plus kit (Zymo Research). RNA (2.0 μg) was oligo-dT primed and reverse-transcribed with the high-capacity RNA-to-cDNA kit (Applied Biosystems). Exon boundaries of *Aurkb* were amplified using TaqMan Assays (Applied Biosystems) as directed by the manufacturer using Beta-2 macroglobulin as a standard. TaqMan Mm03990521_m1 (Rmi2, exon boundary 1-2), Mm01333820_m1 (Rmi1, exon boundary 1-2), Mm00476150_m1 (Blm, exon boundary 18-19), and Mm00437762_m1 (Beta-2 microglobulin). Gene expression was normalized with respect to wild type, with wild-type expression levels considered to be 1.

### Isolation of 4C spermatocytes, determination of CO/NCO frequency and characteristics

Detailed description of methodology and material used in determining CO/NCO frequency, including genomic DNA isolation, determining amplification efficiency of DNA, CO assay, and NCO assay, has been previously described in Frasca *et al*. and Premkumar *et al*. [13, 15].

### Statistical analyses

Results are presented as mean ± standard deviation (SD), and 95% confidence intervals. Statistical analysis was performed using Prism Graph statistical software. Two-tailed unpaired Student’s *t*-test or Mann–Whitney test were used to compare pairwise means. *P* < .05 was considered statistically significant. We used Fisher’s exact test, two-tailed for categorical comparisons.

## Results

### Effect of *Rmi2* deletion in male reproductive tissues

To study the requirement of RMI2 in germ cell development, we generated *Rmi2* germ cell-specific conditional knockout mice. The mutation was created by inserting a Neomycin-LacZ cassette, two FLP recognition sites and a lox P site downstream of exon 1, and an additional loxP site upstream of exon 1 (Supplementary Fig. S1A). A conditional (floxed, FL) allele was created by flipase recombinase expression (FLP) in mice carrying this allele. Subsequent Cre recombinase expression regulated by the promoters of the meiotic specific genes Ddx4 (Ddx4-Cre) resulted in a germline-specific knockout mouse (Supplementary Fig. S1A). Ddx4-Cre (a.k.a. Vasa) activity is directed to male and female germ cells starting at embryonic days 15–18. This allows deletion of the Rmi2 gene as early as in primordial spermatogonia cells (prospermatogonia).

Compared to wild type (0.11 g ± 0.01, *n* = 22 testes, 11 mice), littermate testes in *Rmi2^−/−^* adult mice are significantly smaller; *Rmi2^−/−^* males (0.06 g ± 0.01, *n* = 14 testes, seven mice, *P* < .0001, two-tailed *t*-test) (Fig. 1A).

**Figure 1.**
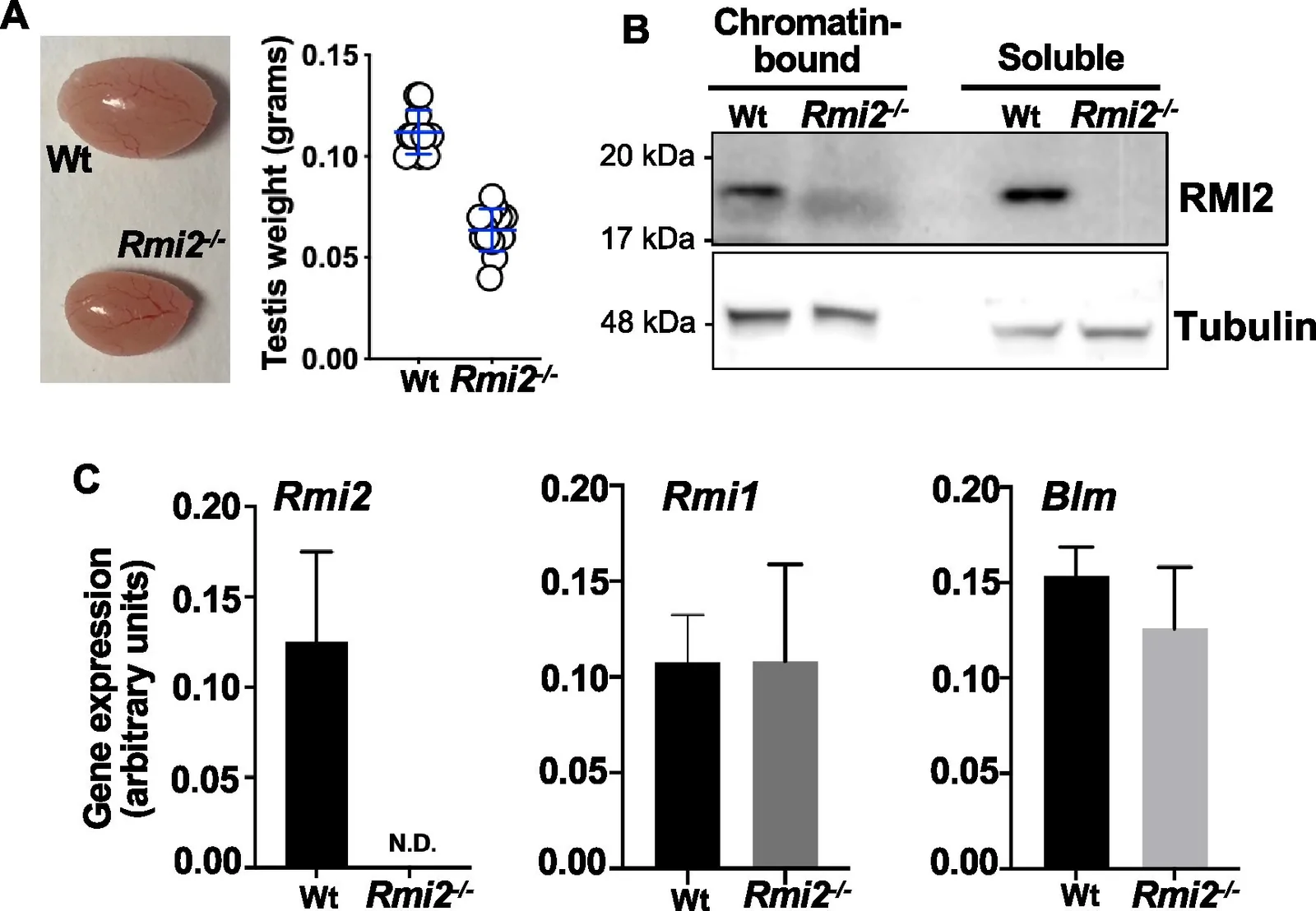
Generation and characterization of a *Rmi2^−/−^*mouse model. (**A**) Testis size comparison between wild type (Wt) and knockout (*Rmi2^−/−^*) mice littermates. The graph on the right shows the differences in weight. Horizontal bars represent mean and standard deviation. (**B**) Western blot showing RMI2 protein in wild type and *Rmi2^−/−^* mice in chromatin-bound and soluble cell fractions. (**C**) Quantitative RT-PCR showing absence of RMI2 in *Rmi2^−/−^* mice and no change in gene expression for Rmi1 and Blm in *Rmi2^−/− ^*mice.

Western blot analysis of whole testis of 2-month-old mice revealed that in contrast to wild-type control, RMI2 was absent in *Rmi2^−/−^*, confirming depletion of RMI2 (Fig. 1B). RT-PCR assessment of *Rmi2, Rmi1*, and *Blm* gene expression confirmed deletion of the *Rmi2* gene and showed no apparent changes in *Rmi1* and *Blm* expression levels after *Rmi2* deletion (Fig. 1C).

We tested a possible effect of RMI2 deletion on BLM or RMI1 binding to chromatin, using enriched fractions of mouse spermatocytes and dividing, soluble, and chromatin-bound protein fractions. Western blotting of the chromatin-bound fraction revealed no apparent changes in either BLM or RMI1 binding to chromatin when wild type and *Rmi2^−/−^* spermatocytes were used (Supplementary Fig. S2A and B).

To assess the reproductive capabilities of male and female *Rmi2^−/−^* mice, we arranged mating experiments in which *Rmi2^−/−^* males were crossed with either *Rmi2*^wt/^*^wt^* or Rmi2^FL/FL^ females, *RMI2^−/−^* males were mated with *RMI2^−/−^* females and contrasted the litter outputs with control crosses between Rmi2^wt/−^ males and Rmi2^FL/FL^ females. Our results show that both male and female Rmi2 knockouts are fertile and produce litter sizes comparable to those of the control (Supplementary Fig. S1B).

In sum, our results show that deletion of *Rmi2* affects certain aspects of testis development, suggesting that RMI2 plays a role during gametogenesis.

### Histological and cellular analysis reveals defects in meiotic chromosome conformation and segregation in *Rmi2^−/−^* mice

RMI2 has been proposed to contribute to the suppression of excess and/or inappropriate recombination events, which, if unresolved, may lead to chromosomal segregation abnormalities. Here, we use histological and cellular approaches to determine the effect of *Rmi2* deletion in meiotic progression and chromosome segregation.

Analysis of paraffin-embedded testes sections stained with hematoxylin and eosin shows that in *Rmi2^−/−^* males, spermatogenesis can progress through meiotic stages and generate spermatozoa (Supplementary Fig. S3A). However, we observed an increase in the number of metaphase–anaphase cell figures in *Rmi2^−/−^* compared to wild type (wild type, 4.76 ± 3.27 metaphase per positive seminiferous tubule, *n* = 24 seminiferous tubules; *Rmi2^−/−^*, 10.08 ± 5.09, *n* = 24, *t*-test, *P* = .0005; data were obtained from three different mice per strain) (Fig. 2A).

**Figure 2.**
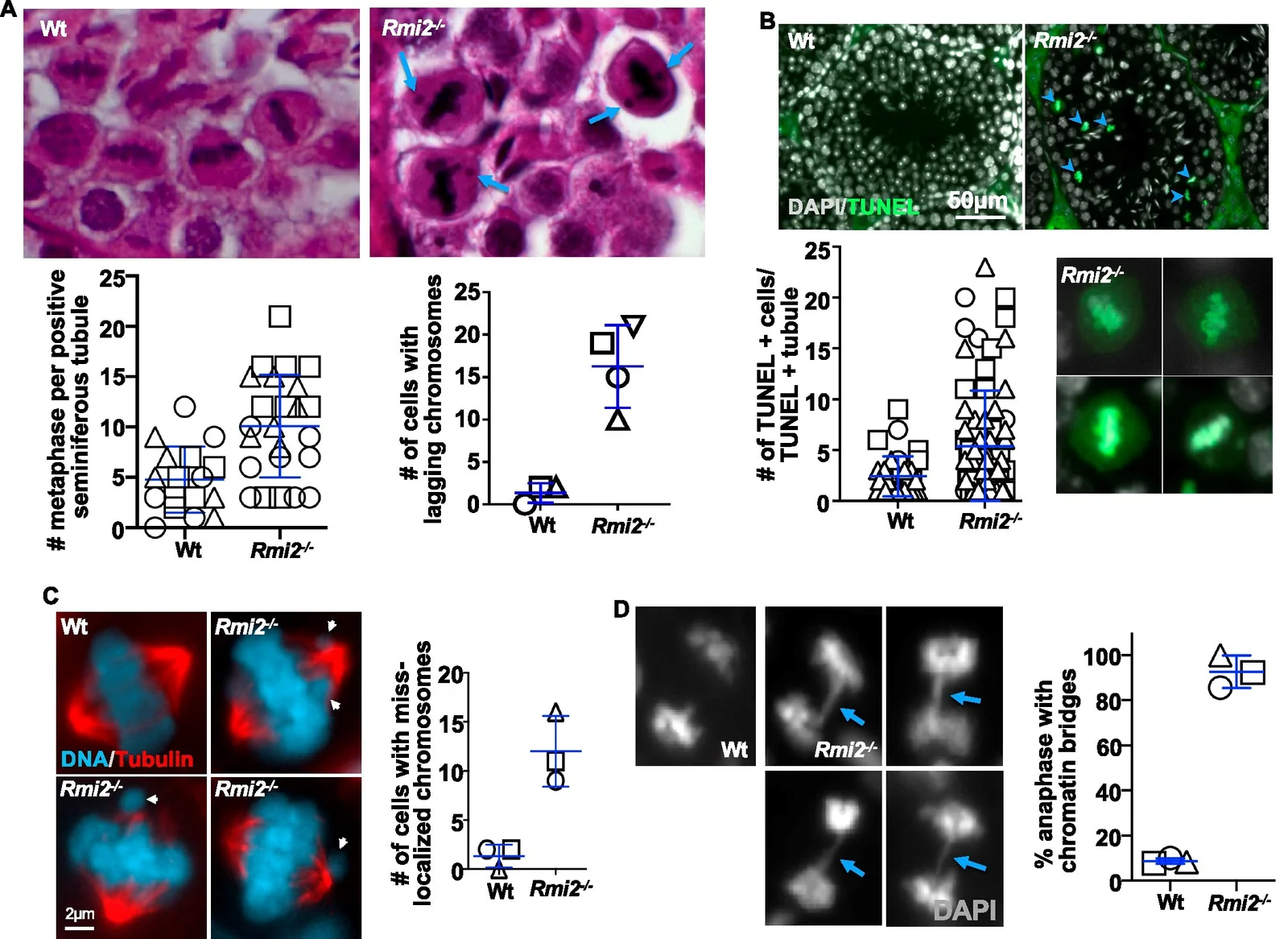
Chromosome conformations and segregation defects in *Rmi2^−/−^* mice. (**A**) Histological analysis of the mice testes. Representative images depicting magnified cells arrested at prophase I exit. The arrows show the presence of metaphase cells with lagging chromosomes. Quantification of metaphase I cells per seminiferous tubule, representing meiotic arrest, and the number of metaphase I cells with lagging chromosomes is also shown. Different symbol types represent results obtained from different mice/biological replicates. (**B**) Terminal deoxynucleotidyl transferase dUTP nick end nuclear labeling (TUNEL) showing an increased number of apoptotic cells in *Rmi2^−/−^* mice compared with control littermates (three mice per group). Quantification of the number of TUNEL-positive cells per TUNEL-positive seminiferous tubule is also shown. (**C**) Representative pictures of metaphase I cells obtained from squashed seminiferous tubules from wild type and *Rmi2^−/−^* mice. The arrows show the presence of lagging chromosomes in metaphase cells. Quantification of the number of cells with chromosomes not aligned at the metaphase I plate is also shown. Different symbol types represent results obtained from different mice/biological replicates. (**D**) Representative images of DAPI-stained anaphase I cells obtained from paraffin-embedded testis sections. The arrows show examples of chromatin bridges. The percentage of anaphase I cells with chromatin bridges counted from three different wild type and three *Rmi2^−/−^* mice is also shown.

Analysis of paraffin-embedded ovary sections stained with hematoxylin and eosin shows no significant differences between wild-type controls and *Rmi2^−/−^* knockouts with respect to the number of observed follicles. This suggests that oogenesis can normally progress through meiotic stages (Supplementary Fig. S3B).

A close look at hematoxylin and eosin stained testis tissue reveals that chromosomes appeared to be normally condensed at metaphase I in *Rmi2^−/−^* cells but, in contrast to wild type, some cells show an abnormal metaphase plate with lagging chromosomes (wild type, 1.33 ± 1.15 cells with lagging chromosomes, *n* = 122 cells counted from three different mice; *Rmi2^−/−^*, 16.25 ± 4.86, *n* = 122 cells counted from four different mice, *t*-test, *P* = .0038) (Fig. 2A, lower right panel).

We then used TUNEL analysis to detect apoptotic cells, possibly revealing the cause of testis weight reduction and cell loss. In contrast to wild type, in which only occasional apoptotic cells are present, seminiferous tubules of 2-month-old *Rmi2^−/−^* mice show more apoptosis in spermatocytes at metaphase (wild type, 2.42 ± 1.96 number of TUNEL positive cell per TUNEL positive seminiferous tubule, *n* = 33; *Rmi2^−/−^*, 5.42 ± 5.42, *n* = 77, Mann–Whitney test, *P* = .0026) (Fig. 2B).

To further understand why *Rmi2* knockout mice show an apparent high rate of abnormal chromosome divisions, we analyzed divisions I and II in spermatocytes in immunostained squashed seminiferous tubules. We used antibodies against tubulin to visualize spindles and DAPI to visualize the chromosomes. Most primary (meiotic division I) spermatocytes from control males exhibited normally condensed chromosomes congressing around the spindle equator (wild type, 1.33 ± 1.15 cells with mis-localized chromosomes, *n* = 75 from three different mice). Chromosome aberrations in *Rmi2^−/−^* were evident as different lagging chromosome conformations were observed outside of the metaphase plate (12.00 ± 3.60 cells with mis-localized chromosomes, *n* = 75 from three different mice; *t*-test, *P* = .008) (Fig. 2C).

Unresolved recombination JMs, some of which may contain or can be converted into hemicatenane-like structures, are proposed to be resolved by the BTRR complex [47, 48] (reviewed in Sarlos *et al*. [48]). BTRR is involved in resolving chromosome bridges by unwinding and separating entangled DNA strands. A chromosome bridge forms at anaphase of meiosis when chromatids are not free to separate and form a bridge between two chromosomes, segregating to the opposite poles. Based on these findings, we reasoned that RMI2 may participate in resolving chromatin bridges observed during anaphase of meiosis I. If this is the case, *Rmi2^−/−^* spermatocytes should show an increase in chromatin bridges in prophase I spermatocytes undergoing the first meiotic division. Supporting this hypothesis, we observed that the number of meiotic anaphase I cells with chromatin bridges significantly increased in *Rmi2^−/−^* (92.63% ± 7.2 anaphase cells with chromatin bridges, *n* = 24 obtained from three different mice) compared to wild type (8.67 ± 1.19, *n* = 35 obtained from three different mice; *t*-test, *P* = .0001) (Fig. 2D). We speculate that the observed chromatin bridges are likely to originate from unrepaired recombination or topological intermediates formed during prophase I, and note that the BTRR complex may function in their eventual resolution if they persist into anaphase I. The high frequency of anaphase bridges observed in *Rmi2^−/−^* spermatocytes may indicate the presence of a compensatory or alternative pathway capable of resolving the remaining DNA catenates. Such a mechanism could allow a subset of spermatocytes to successfully complete meiosis despite widespread chromatin abnormalities. Consequently, even though most cells exhibit defects, sufficient numbers may progress to produce functional gametes, thereby preserving overall fertility.

In summary, our analysis suggests that spermatogenesis appears normal until the end of prophase I, when a subset of spermatocytes exhibits defects in metaphase–anaphase I. These spermatocytes exhibit aberrant chromosome conformation and ultimately undergo apoptosis, likely due to unresolved DNA recombination intermediates.

### Cytological markers suggest defective processing of intermediate stages of recombination in *Rmi2^−/−^* spermatocytes

To assess whether recombination was affected by *Rmi2* deletion, we immunostained mouse spermatocyte chromosome spreads obtained from *Rmi2^−/−^* and wild-type littermates with marker proteins representative of different stages in the recombination pathway.

We used antibodies against the SYCP3 protein to reveal meiotic chromosome axes and the stage of prophase I. γH2AX marks double-strand breaks [49], and in mouse spermatocytes, the entire nucleus in leptotene–zygotene spermatocytes remains at the sex body in pachytene and diplotene cells [10]. γH2AX immunostaining analysis shows no apparent differences between wild-type and knockout spermatocytes (e.g. remnant γH2AX signal in autosomes) (Supplementary Fig. S4A).

MLH1 is involved in the resolution of Holliday junction structures (a later intermediate in the recombination pathway) [50, 51] and is widely used as a marker of the CO site [52]. Wild type and *Rmi2^−/−^* spermatocyte analysis shows no difference in MLH1 foci number per cell (wild type, 22.85 ± 2.55 foci per cell; *Rmi2^−/−^*, 23.82 ± 2.76, *n* = 60) (Fig. 3A). We quantified CO events per chromosome (Supplementary Fig. S4B) and assessed MLH1 foci distribution as a proxy for CO interference (Supplementary Fig. S4C). In both analyses, no significant differences were detected between *Rmi2^−/−^* mutants and wild-type controls.

**Figure 3.**
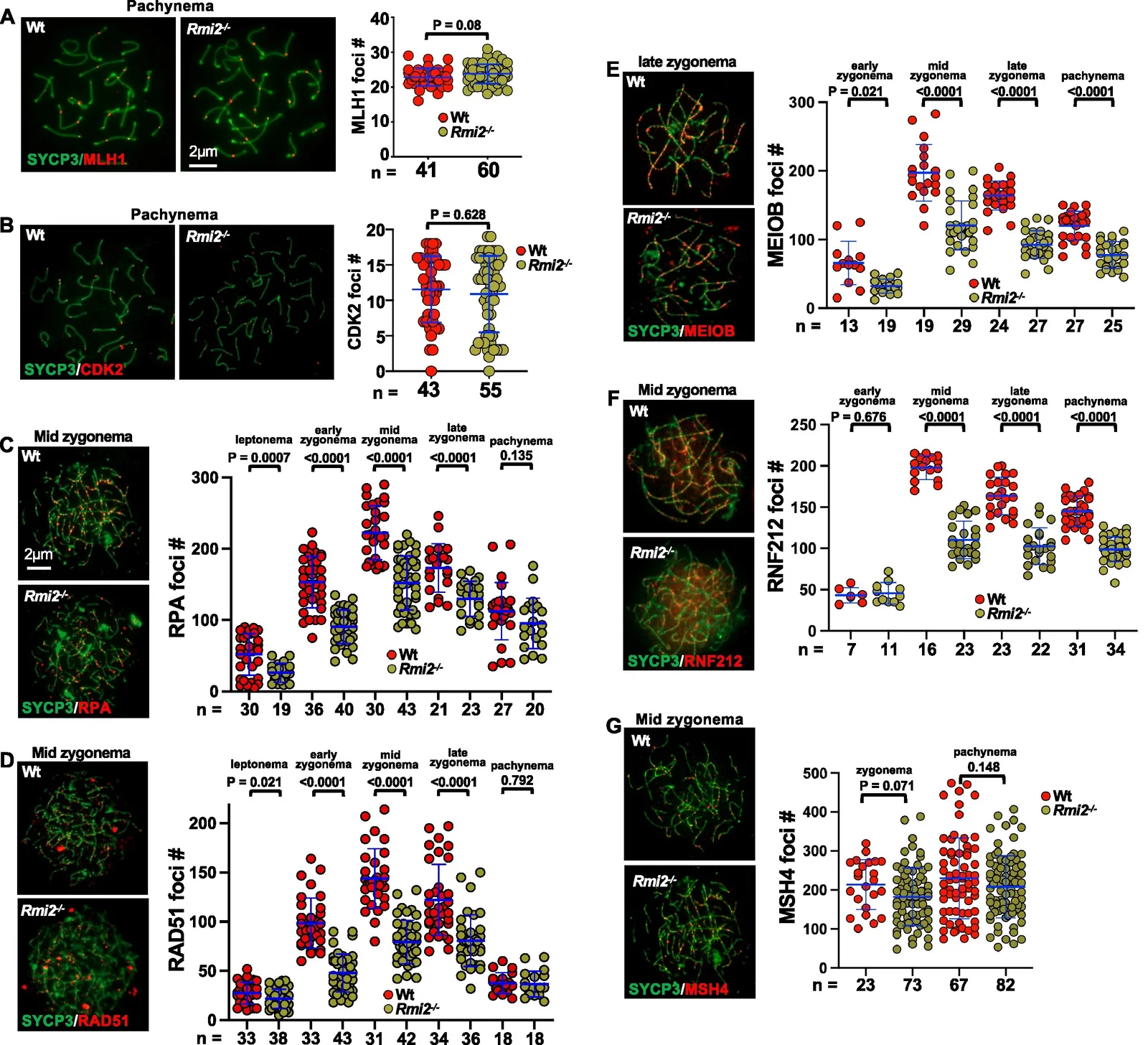
*Rmi2^−/−^* spermatocytes show defects in the processing of recombination intermediates. Representative images of prophase I spermatocyte chromosome spreads obtained from wild-type (Wt) and *Rmi2^−/−^* mice. Mice spreads were stained with SYCP3 antibody (to mark the chromosome core) and antibodies against different cytological markers of double-strand break repair and recombination intermediates. Chromosome spreads showing typical immunostaining and quantification of the number of foci per nucleus for cytological markers of late [MLH1 (**A**) and CDK2 (**B**)] and of mid stages of recombination; RPA (**C**), RAD51 (**D**), MEIOB (**E**), RNF212 (**F**), and MSH4 (**G**). Magnification bar in panel (A) corresponds to all spermatocytes in panels (A) and (B). Magnification bar in panel (C) corresponds to all spermatocytes in panels (C)–(G). The number of cells quantified (*n*) and the *P*-value corresponding to the indicated statistical comparisons are represented at the bottom and top of each panel, respectively. For all conditions, quantified cells were obtained from three independent mice (biological replicates).

CDK2 localizes to late recombination nodules, and it is suggested to play an important role in regulating MLH1 foci number [53]. In agreement with MLH1 results, analysis of CDK2 foci number shows no significant difference between wild type and *Rmi2^−/−^* mice (wild type, 11.56 ± 4.67 foci per positive cell; *Rmi2^−/−^*, 10.89 ± 5.38) (Fig. 3B).

We then assessed the number of bivalents per nuclei revealed by air-dried metaphase spreads in 20-day-old mice (wild type, 19.33 ± 1.24 foci per cell, *n* = 30 counted cells; *Rmi2^−/−^*, 19.43 ± 1.55, *n* = 44, Mann–Whitney test, *P* = .26), 2-months-old mice (wild type, 19.56 ± 0.67 foci per cell, *n* = 32 counted cells; *Rmi2^−/−^* 19.86 ± 0.895, *n* = 51 Mann–Whitney test, *P* = .051) (Supplementary Fig. S4D and E, respectively), or in 2-months-old mice expressing two additional copies of SPO11 (wild type 19.67 ± 0.76 foci per cell, *n* = 30; *Rmi2^−/−^*^/tg++^ 19.80 ± 0.88, *n* = 30, *t*-test, *P* = .53) (Supplementary Fig. S4F). In none of these conditions was the number of bivalents per nucleus significantly different between wild-type control littermates and *Rmi2^−/−^* knockout spermatocytes.

The γH2AX and MLH1 results suggest that *Rmi2* deletion has no gross defect/delay in DSB repair, and that the final products of CO recombination leading to chiasma formation are normal.

We then reasoned that whether, as suggested in other experimental systems, the BTRR complex and RMI2 activity are required for the resolution of JMs, part of the intermediate DNA recombination metabolism, we should observe differences in cytological markers indicative of these stages. To test this, we performed a comparative immunostaining analysis in *Rmi2^−/−^* and wild type using typical marker proteins for intermediate stages of the recombination pathway. SPO11-induced DSBs are immediately resected to expose 3′ ssDNA overhangs, which are bound by the RPA complex to protect the integrity of this recombination intermediate. Then, RAD51 displaces RPA and, together with DMC1, forms a nucleoprotein filament capable of performing a homology search on the intact homologous chromosome [2]. As expected, we observed an increase, followed by a decrease, in the number of RPA foci per cell as cells progressed through meiosis. In wild type and *Rmi2^−/−^*, a peak in the number of RPA was observed at mid zygonema. However, we observed a significant change in the number of RPA foci per cell in *Rmi2^−/−^* compared to wild type in leptonema (wt, 52.10 ± 29.12 versus *Rmi2^−/−^*, 26.21 ± 13.43), early zygonema (wt, 153.2 ± 36.16 versus *Rmi2^−/−^*, 90.73 ± 23.35), mid zygonema (wt, 222.4 ± 37.91 versus *Rmi2^−/−^*, 152.2 ± 37.95), and late zygonema (wt, 173.1 ± 34.08 versus *Rmi2^−/−^*, 130.2 ± 24.52). No significant difference was observed in pachynema (wt, 112.6 ± 40.17 versus *Rmi2^−/−^*, 95.50 ± 35.31) (Fig. 3C). A similar behavior was observed in the number of RAD51 foci per cell as the cell progressed through meiosis. A significant change in the number of RAD51 foci per cell in *Rmi2^−/−^* compared to wild type was observed in leptonema (wt, 27.42 ± 10.93 versus *Rmi2^−/−^*, 21.66 ± 9.71), early zygonema (wt, 98.61 ± 25.50 versus *Rmi2^−/−^*, 47.88 ± 19.24), mid zygonema (wt, 143.8 ± 30.18 versus *Rmi2^−/−^*, 79.33 ± 22.06), and late zygonema (wt, 122.1 ± 36.16 versus *Rmi2^−/−^*, 80.72 ± 26.16). No significant difference was observed in pachynema (wt, 37.72 ± 10.57 versus *Rmi2^−/−^*, 36.67 ± 13.06) (Fig. 3D).

MEIOB is a conserved meiotic paralog of RPA, the absence of which in mouse spermatocytes leads to sterility as a result of the accumulation of unrepaired DSBs during meiotic prophase I [54]. Deletion of *Rmi2* resulted in a significantly lower number of MEIOB foci in mid zygonema (wt, 197.3 ± 41.16 versus *Rmi2^−/−^*, 120.9 ± 35.62), late zygonema (wt, 164.00 ± 21.24 versus *Rmi2^−/−^*, 92.74 ± 20.20), and pachynema (wt, 120.2 ± 21.05 versus *Rmi2^−/−^*, 77.36 ± 19.52) (Fig. 3E).

RNF212 has been proposed to stabilize pro-crossover factors that directly mediate the DNA events of crossing over [55, 56]. RNF212 activity results in the loss of MutSγ from most recombination sites, except those at prospective CO sites, which then accumulate CO-specific factors, such as the MutLγ endonuclease [50, 51]. Deletion of *Rmi2* resulted in a significantly lower number of RNF212 foci in mid zygonema (wt, 198.0 ± 14.28 versus *Rmi2^−/−^*, 110.3 ± 22.62), late zygonema (wt, 163.60 ± 22.58 versus *Rmi2^−/−^*, 102.6 ± 22.21), and pachynema (wt, 145.10 ± 18.35 versus *Rmi2^−/−^*, 98.76 ± 15.14) (Fig. 3F).

MSH4 is part of the MutSγ complex (MSH4–MSH5), which can bind JMs such as D-loop and Holliday junction intermediates [51, 57], and is inferred to protect them from dissociation by the BTRR complex. Compared to wild-type spermatocytes, *Rmi2^−/−^* cells show no significant difference in the number of MSH4 foci per cell (zygonema wt, 213.5 ± 63.93; *Rmi2^−/−^*,182.3 ± 73.60 and pachynema wt, 229.9 ± 103.6; *Rmi2^−/−^*, 208.00 ± 79.90) (Fig. 3G).

Our observations suggest that the loss of RMI2 results in abnormal processing of recombination intermediates, leading to CO formation. We speculate that CO homeostasis [58]—the maintenance of CO number despite fluctuations in the number of earlier recombination intermediates—may compensate for the moderate imbalance in recombination intermediates formation, resulting in an apparent normal number and placement of COs per chromosome.

### Imbalanced CO/NCO formation in *Rmi2****^−^****^/**−**^* spermatocytes

To directly assess the effect of *Rmi2* deletion on recombination outcomes, we monitored the formation of COs and NCOs at the well-characterized *59.5* hotspot located on chromosome 19 and the *A3* hotspot located on chromosome 1 in wild-type and *Rmi2^−/−^*spermatocytes. This is a highly sensitive assay that enables fine-scale mapping of recombination events at highly active recombination hotspots in F1 hybrids derived from the inbred DBA/2J (D) and C57BL6/J (B) mouse strain [13]. The *59.5* hotspot is asymmetric, with a higher frequency of meiotic DSBs on the B than on the D chromosome [46]. By contrast, *A3* is symmetric and is equally likely to receive a break on either parental chromosome [59]. These hotspots also differ in that *59.5* is highly enriched for MutLγ-dependent COs, while *A3* is enriched for NCOs. As a control, we compared results with outcomes obtained after deletion of recombination factors known to affect crossing over, *Mlh3*^−/−^ and *Exo1*^−/−^ mutant spermatocytes [15].

To directly compare genotypes, we isolated late 4C spermatocytes (mostly diplotene cells) by fluorescence-activated cell sorting at the end of meiotic prophase I [13, 15, 60]. In our experiments (Fig. 4A), a low concentration of DNA extracted from late 4C cells is used to seed parallel reactions. The entire hotspot is amplified using two consecutive nested PCRs, with allele-specific primers on one side and a universal primer that recognizes both parental chromosomes equally on the other side (e.g. B to U). The amplification is non-selective, such that the ratio of non-recombinant (parental), COs, and NCOs is preserved. In such a way, the frequency of repair products can be compared across genotypes (Fig. 4B). There are three recombination outcomes observed with this assay: (i) COs; (ii) singleton NCOs that involve gene conversion to the donor genotype on the cut chromosome of only a single polymorphism; and (iii) co-converted NCOs that involve conversion of multiple, often adjacent polymorphisms. Loss of RMI2 resulted in a significant shift in pathway usage, with 67% fewer COs (Fig. 4B and Supplementary Fig. S5) and 380% more NCOs at *59.5* (*P* < .0001 for both, Fisher’s exact test, two-tailed). The higher frequency of singleton NCOs was like other mutants that fail to mature COs efficiently, like *Mlh3^−/−^* and *Exo1^−/-^* (Fig. 4B) [15]. The lower CO frequency and compensatory increase in NCOs at *59.5* may be related to fewer JMs (e.g. MSH4) observed in *Rmi2^−/−^*.

**Figure 4.**
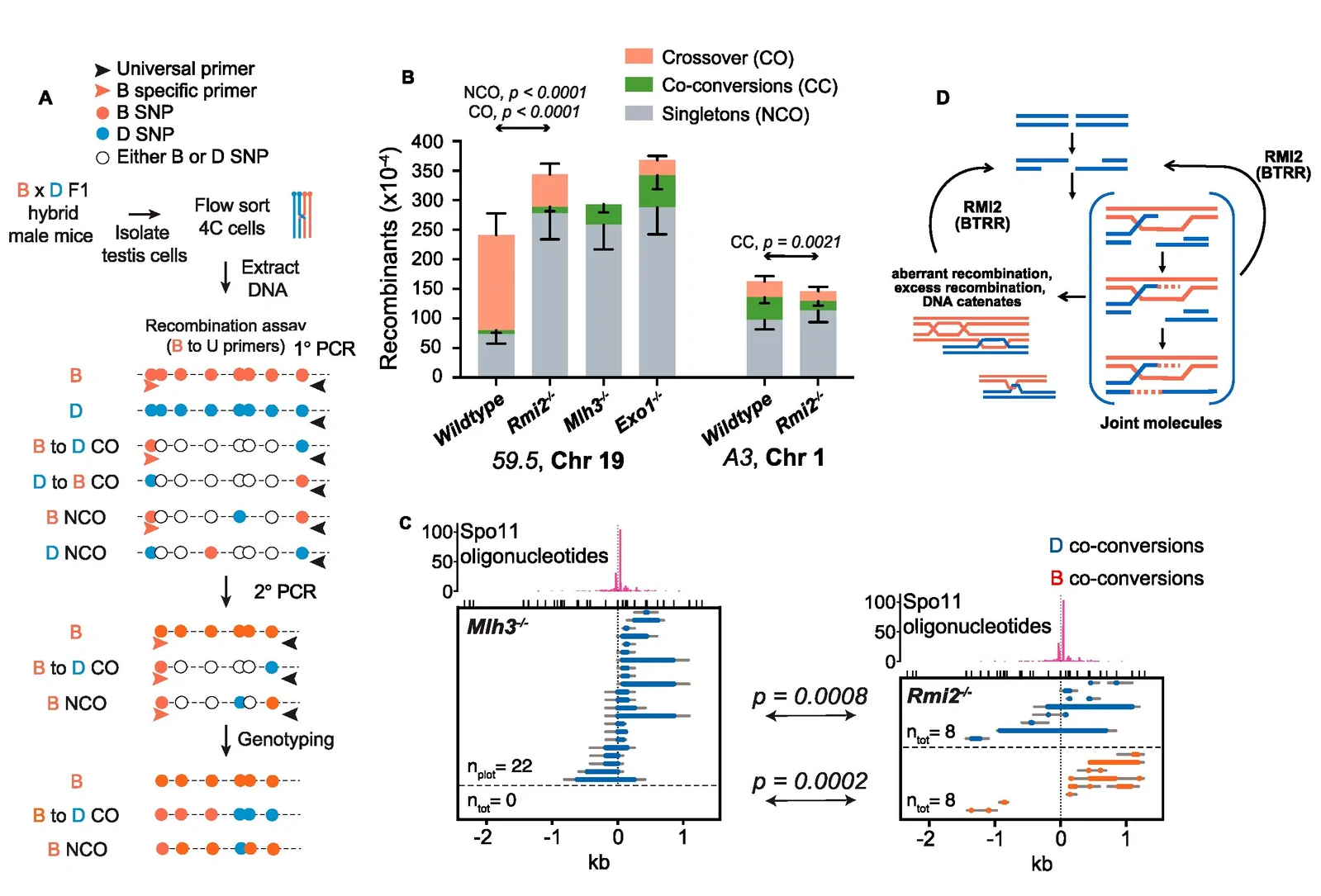
CO and NCO outcome in *Rmi2^−/−^* mice. (**A**) Schematic showing the PCR-based strategy used to amplify recombinants. Represented are possible outcomes, polymorphisms on the B and D chromosomes, and specific and universal primers. (**B**) Frequency of recombinants (CO, NCO, co-converted NCO) observed per indicated genotype at *59.5* and *A3* hotspots. (**C**) The distribution of co-converted NCOs on the cut (B) and donor (C) chromosomes at *59.5* in spermatocytes lacking MLH3 (left) and RMI2 (right). (**D**) Schematics of the proposed model for RMI2 and BTRR function in eukaryotes meiosis.

While the frequency of co-converted NCOs was unchanged at *59.5* (Fig. 4B), the distribution was markedly different (Fig. 4C). Notably, most co-converted NCOs at *59.5* are derived from CO precursors that have failed to mature into COs and are dissolved into NCOs, many of them involving longer gene conversion tracts associated with CO intermediate precursors [15]. Despite a high frequency of co-converted NCOs in CO maturation mutants, we only observe them on the cut chromosome (B). In contrast, in the absence of RMI2, we see an equal number of co-converted NCOs on both the donor and cut chromosomes. In addition, on the cut chromosome, these co-converted NCOs are distributed throughout the hotspot and do not cluster around the hotspot center. The hotspot center corresponds to the 3′ ends of the SPO11-dependent DSBs (compare with *Mlh3^−/−^*). Markedly, on the donor chromosome, the co-converted NCOs are shifted to the far right and left of the hotspot. The COs that fail to form at *59.5* in the absence of RMI2 are likely being dissolved into co-converted NCOs, but with significantly different branch migration properties than observed in the absence of MLH3 or EXO1 [15].

At *A3*, the overall MLH3-dependent CO frequency is much lower than at *59.5*, and we failed to see a significant reduction in COs (Fig. 4B). However, we do see fewer co-converted NCOs between *Rmi2^−/−^* and wild type control (*P* = .0021). Importantly, most co-converted NCOs at *A3* are not associated with failed CO maturation but instead are likely derived from synthesis-dependent strand annealing. This finding supports the possible model that loss of RMI2 results in altered branch migration of both CO and NCO precursor intermediates.

We performed a comparative analysis in *Rmi2^−/−^* and *Blm^−/−^* knockout mice (Supplementary Fig. S5). Our results show that the CO recombination frequencies of *Rmi2^−/−^* and *Blm^−/− ^*knockout mice are statistically significantly different from wild type (*P* < .0001, Chi-squared with Yates correction, two-tailed) but not significantly different from each other (*P* = .113). These results agree with a model in which RMI2 is critical for BLM’s pro-CO function.

## Discussion

The BTRR complex, consisting of the BLM helicase, TOP3A, RMI1, and RMI2, plays a crucial role in maintaining genome stability by resolving excess or aberrant recombination intermediates that arise during homologous recombination. While BLM, TOP3A, and RMI1 are widely conserved across eukaryotic species, RMI2 is absent in simple model organisms, suggesting that its function may be important in organisms with greater genomic complexity.

In mitotic cells, CO formation is actively suppressed to prevent inappropriate sister chromatid exchange. The BTRR complex plays a central role in this process by promoting the dissolution of joint DNA molecules into NCO products through a decatenation mechanism [37–39]. Within this complex, RMI2 is thought to simply stabilize RMI1, with its loss leading to elevated sister chromatid exchange, as demonstrated in chicken DT40 cells following *Rmi2* knockdown [61]. In mammals, the broader relevance of RMI2 to development and disease susceptibility has been underscored by the identification of a homozygous *Rmi2* deletion in two male siblings exhibiting features of Bloom syndrome [43]. Supporting these findings, CRISPR–Cas9-mediated disruption of RMI2 in human fibroblasts suggests the hyper-recombination phenotype and mitotic abnormalities observed in patient-derived cells [43]. Accordingly, the Rmi2 knockout mouse model we generated may provide a valuable system for disease modeling. Consistent with this, our findings in Rmi2-deficient mice expand upon several phenotypes observed in human patients (discussed below).

Motivated by prior evidence implicating RMI2 in regulating the BTRR complex and recombination outcomes, we investigated RMI2’s contribution to meiotic recombination and male gametogenesis. To this end, we generated an RMI2-deficient mouse model and analyzed recombination-associated phenotypes at tissue, cellular, and molecular levels. Loss of RMI2 resulted in marked meiotic abnormalities, including increased meiotic arrest at the transition from prophase I to the first meiotic division and elevated germ cell apoptosis. Further analysis revealed pronounced chromosome segregation defects, characterized by increased frequencies of lagging chromosomes during metaphase I and the formation of chromatin bridges in anaphase I in mouse spermatocytes. Although these defects were frequent, the retained fertility of *Rmi2^−/−^* mutant mice suggests that compensatory or alternative mechanisms may mitigate their impact, allowing a subset of spermatocytes to complete meiosis and produce functional gametes.

Although the resolution of joint DNA molecules during meiosis is often attributed to structure-selective endonucleases, dissolution mediated by the TOP3α–RMI1 complex is essential for resolving complex recombination intermediates and ensuring faithful chromosome segregation. Studies in yeast, *Caenorhabditis elegans*, and *Arabidopsis thaliana* have suggested that the activity of the BLM helicase generates hemicatenane structures during the dissolution of dHJs [62], which are subsequently resolved by TOP3α in concert with RMI1 and RMI2 [42, 61, 63–65]. *In vitro* analyses have shown that RMI1 and RMI2 form a non-catalytic sub-complex that is critical for the efficient processing of these recombination intermediates [66, 67]. Consistent with this, the increased incidence of abnormal chromosome conformations and segregation defects observed in *Rmi2*^−/*−*^mouse spermatocytes parallels the elevated frequency of bulky DNA bridges reported in *C. elegans* RMIF-2 mutants, the ortholog of RMI2 [44]. Similarly, independent of Sgs1 function, yeast Top3-Rmi1 has been shown to resolve recombination-dependent chromosome entanglements, thereby promoting proper meiotic chromosome segregation at anaphase [24]. Collectively, our findings support a common model in which RMI2 facilitates TOP3α- and/or RMI1-dependent resolution of topological links between homologous chromosomes. In the absence of RMI2, unresolved entanglements likely persist, resulting in aberrant chromosome architecture and defective segregation during meiosis (Fig. 4D).

The participation of mouse RMI2 in the metabolism of recombination JMs has been apparent by the analysis of cytological markers of the intermediate state of recombination, such as RPA, MEIOB, MSH4, and RNF212. Although the phenotype observed in *Rmi2* mutant spermatocytes is moderate, the consistent reduction in the number of foci observed agrees with a regulatory function proposed for RMI2. This RMI2 engagement with JMs is consistent with a BTRR function observed in other organisms. For example, analyses of the yeast STR complex (Sgs1 helicase–topoisomerase–Rmi1) have revealed that its meiotic functions include a role in D-loop reversion *in vivo* [23, 24], which has also been shown *in vitro* [68]. The reduction in early recombination intermediates observed in *Rmi2* mutant spermatocytes, as indicated by decreased RAD51 foci, is not necessarily incompatible with the maintenance of normal MLH1-dependent CO numbers. Defective homolog engagement can stimulate additional DSB formation, promoting interhomolog interactions and synapsis [69, 70], thereby replenishing the pool of recombination substrates. Furthermore, CO homeostasis can preserve CO numbers despite alterations in DSB frequency or early recombination intermediates by reallocating repair events between CO and NCO pathways. Given the established roles of the BTRR complex in D-loop reversal and recombination intermediate processing, loss of RMI2 likely reduces the efficiency of homolog engagement and CO formation. The maintenance of normal MLH1 counts and, ultimately, fertility therefore highlights the capacity of compensatory meiotic mechanisms to buffer perturbations in recombination pathway function.

To define the role of RMI2 in recombination outcomes at the molecular level, we monitored the formation of COs and NCOs using a sensitive analysis at the *59.5* and *A3* hotspots in wild type and *Rmi2^−/−^* spermatocytes. The results revealed an imbalance in CO/NCO formation with *Rmi2^−/−^* showing a marked decrease in COs and a concomitant increase in NCOs. These findings underscore the pro–crossover role of BTRR in promoting interhomolog JMs that can be stabilized by MutSγ. In *C. elegans*, the functional RMI2 homolog RMIF-2 localizes to recombination foci during meiotic prophase I, and its concentration into recombination foci is mutually dependent on other BTRR complex proteins. Comparative analysis of the RMIF-2 and RMH-1 mutant phenotypes revealed numerous commonalities, including in regulating CO formation and directing COs toward chromosome arms [31, 33, 40].

Our results suggest previously unrecognized characteristics in the processing of recombination intermediates and in the function of mammalian RMI2. For example, the prevalence of short NCO tracts in Rmi2–deficient mouse spermatocytes indicates that synthesis–dependent strand annealing remains active and is not suppressed. This contrasts sharply with budding yeast, where NCO in *sgs1, rmi1*, or *top3* mutants are predominantly generated through structure–selective nuclease resolution. We also observed alterations in gene conversion patterns that can be attributed to differences in branch migration in the absence of RMI2. At the *59.5* hotspot, *Rmi2^−/−^* spermatocytes, co-converted NCOs occurred equally on both cut and donor chromosomes and extended beyond the DSB center into distal regions of the hotspot, in contrast to the restricted, center-focused tracts seen in wild type, *Mlh3^−/−^*, or *Exo1^−/−^* cells. On the donor chromosome, conversion tracts were shifted toward the periphery of the hotspot, suggesting excessive or unregulated branch migration of strand invasion intermediates. At the *A3* hotspot, loss of RMI2 selectively reduced co-converted NCOs, consistent with a broader role for RMI2 in limiting inappropriate branch migration regardless of the recombination pathway. These results provide unprecedented resolution of recombination outcomes and associated gene conversion patterns, enabling a direct link between distinct joint-molecule intermediates and their molecular signatures, and offering substantial novel mechanistic insights into RMI2 function. Collectively, these findings support a model in which RMI2 restricts branch migration of meiotic recombination intermediates, promoting controlled heteroduplex formation and proper CO maturation.

The prevailing model in both somatic and meiotic contexts posits that RMI2 functions primarily as a scaffold protein, stabilizing other members of the BTRR complex and thereby playing a relatively minor regulatory role. In contrast, our results suggest that RMI2 exerts an important influence on the choice of recombination pathway and in maintaining balanced CO and NCO outcomes. Taken together, integrating observations across different organisms reveals that the BTRR complex controls balanced CO/NCO formation during meiosis [23–26, 32, 34–36], with mammalian RMI2 playing an important, previously unrecognized regulatory role (Fig. 4D).

## Conclusion

The accurate separation of chromosomes during gamete formation relies on carefully controlled recombination-mediated DNA exchanges called crossovers. A protein complex known as the BTRR complex regulates these exchanges in normal body cells, but its role in reproductive cells is less clear. This study examines RMI2’s role in mouse sperm development using a knockout model. Loss of RMI2 causes defects in meiotic cell divisions, increased cell death, and severe chromosomal abnormalities. At the molecular level, our study provides high-resolution insight into the processing of meiotic joint molecules. We demonstrate that RMI2 plays an active, previously underappreciated role in regulating the balance among recombination pathways, the proper formation of COs, and chromosome architecture. Together, these findings redefine RMI2 as a key determinant of recombination intermediate processing and genome stability during mammalian meiosis.

## Supplementary Material

gkag950_Supplemental_Files

## Data Availability

The data underlying this article are available in the article and in its online Supplementary data.
