# Supplementary material for: RMI2 regulates recombination intermediate metabolism to ensure proper chromosome segregation in mammalian meiosis
Source: Nucleic Acids Res. 2026 Oct 5;54(18):gkag950. doi: 10.1093/nar/gkag950 (PMC13635748; doi:10.1093/nar/gkag950)
Supplement: Supplementary file 1 [file gkag950_supplemental_files.zip › Paper draftsupplementaryonly.docx]

**Supplementary Figure Legends**

**Figure S1. Rmi2^-/-^ mice design and fertility test. (A)** Schematics showing the mouse conditional knockout strategy. **(B)** crosses and breeding results.

**Figure S2. Effect of Rmi2 deletion in BLM and RMI1 binding to chromatin.** **(A)** Western blots showing BLM or RMI1 immunosignal from chromatin-bound proteins obtained from wild type and *Rmi2^-/-^* meiotic spermatocytes. Tubulin is used as a loading control. (1-4) represent individual mice tested. Quantification of band intensity is also shown. **(B)** Quantitation corresponding to BLM, RMI1, and tubulin band density in western blots shown in A. To determine changes in BLM or RMI1 immunosignal intensity was normalized to the corresponding tubulin values.

**Figure S3. Gametogenesis progression in *Rmi2^-/-^ mice*.** **(A)** Histological analysis of wild type and *Rmi2^-/-^* mice testes. The lower panel shows magnified seminiferous tubules. The arrows show cells at metaphase. **(B)** Histological analysis of wild type and *Rmi2^-/-^* mice ovaries. Quantitation of the number of follicles per ovary section is also shown (wild type 5.38 ± 1.06; *Rmi2^-/-^* 5.62 ± 1.18, P = 0.66 two-tailed t-test).

**Figure S4. Characterization of crossover formation in *Rmi2^-/-^*** **mice. (A)** Representative mouse spermatocytes immunostained with SYCP3 (to mark chromosome axis) and ƔH2AX (a marker of double-strand breaks). M. zygonema: mid-zygonema and L. zygonema: late zygonema. **(B)** Quantification of the number of 0, 1, 2, and more than 2 MLH1-marked crossovers (CO) per chromosome observed in wild-type and *Rmi2^-/-^* spermatocytes obtained from three different mice. **(C)** Distance distribution between two MLH1 foci on meiotic chromosome cores (SYCP3) obtained for wild type and *Rmi2^-/-^* spermatocytes. **(D),** **(E),** and **(F)** Representative metaphase spreads showing an equal number of bivalents per nucleus in wild-type spermatocytes and *Rmi2^-/-^* spermatocytes obtained from 20-day-old (D), 2-month-old (E), and 2-month-old mice expressing two extra copies of SPO11 (transgenic SPO11 mice) (F). The number of bivalents per cell is also shown.

**Figure S5. Comparison of CO and NCO outcome in wild type, Rmi2^-/-^**, **and Blm^-/-^ mice.** Histogram showing CO exchange point frequency in centimorgans per Megabase (cM/Mb) and distribution of crossovers around the hotspot center set at 0 kb for wild type, *Rmi2^-/-^*, and *Blm^-/-^* mice. The area under the curve represents the total CO frequency. Vertical dotted lines mark the average location of CO exchange points based on the cumulative distribution. The bottom graph shows a histogram of *Rmi2^-/-^* with a scale adjustment. Chi-square with Yates correction was used to analyze statistical significance between samples.

**Table S1.** Antibodies used in this study.
