# Supplementary material for: RMI2 regulates recombination intermediate metabolism to ensure proper chromosome segregation in mammalian meiosis
Source: Nucleic Acids Res. 2026 Oct 5;54(18):gkag950. doi: 10.1093/nar/gkag950 (PMC13635748; doi:10.1093/nar/gkag950)
Supplement: Supplementary file 1 [file gkag950_supplemental_files.zip › Table S1 Antibodies.docx]

**Table S1**

**Primary antibodies**

| Antibody | Source | Western blot dilution | Immuno-labeling dilution |
| --- | --- | --- | --- |
| SYCP3 | Our lab. ref: PMC5962103 |  | 1:300 |
| γH2AX | Millipore, 05-636 |  | 1:1,000 |
| MLH1 | BD Pharmingen, 550838 |  | 1:30 |
| CDK2 | Santa Cruz, SC6248 |  | 1:100 |
| RPA | Cell signaling, 2208 |  | 1:100 |
| RAD51 | Santa Cruz, SC8349 |  | 1:100 |
| MEIOB | Millipore, ABE 1414 |  | 1:300 |
| MSH4 | Abcam, ab58666 |  | 1:300 |
| RNF212 | Neil Hunter lab. ref: PMC4245152 |  | 1:250 |
| BLM | abcam, ab2179 | 1:500 |  |
| RMI1 | MyBioSource, MBS129174 | 1:500 |  |
| RMI2 | Invitrogen, PA595632 | 1:500 |  |
| tubulin | Proteintech, 66031-1-Ig | 1:5,000 |  |
